# Facile Pressure-Sensitive Capacitive Touch Keypad for a Green Intelligent Human–Machine Interface

**DOI:** 10.3390/s22218113

**Published:** 2022-10-23

**Authors:** Muhammad Shumail Malik, Muhammad Hamza Zulfiqar, Muhammad Atif Khan, Muhammad Qasim Mehmood, Yehia Massoud

**Affiliations:** 1 Department of Biomedical Engineering, Narowal Campus, University of Engineering and Technology, Lahore 54890, Pakistan; 2 Innovative Technologies Laboratories (ITL), King Abdullah University of Science and Technology (KAUST), Thuwal 23955, Saudi Arabia

**Keywords:** capacitive pressure sensors, touch keypad, intelligent HMI, green, graphite-on-paper

## Abstract

There is a great demand for human–machine interfaces (HMIs) in emerging electronics applications. However, commercially available plastic-based HMIs are primarily rigid, application-specific, and hard to recycle and dispose of due to their non-biodegradability. This results in electronic and plastic waste, potentially damaging the environment by ending up in landfills and water resources. This work presents a green, capacitive pressure-sensitive (CPS), touch sensor-based keypad as a disposable, wireless, and intelligent HMI to mitigate these problems. The CPS touch keypads were fabricated through a facile green fabrication process by direct writing of graphite-on-paper, using readily available materials such as paper and pencils, etc. The interdigitated capacitive (IDC) touch sensors were optimized by analyzing the number of electrode fingers, dimensions, and spacing between the electrode fingers. The CPS touch keypad was customized to wirelessly control a robotic arm’s movements based on the touch input. A low-pressure touch allows slow-speed robotic arm movement for precision movements, and a high-pressure touch allows high-speed robotic arm movement to cover the large movements quickly. The green CPS touch keypad, as a disposable wireless HMI, has the potential to enforce a circular economy by mitigating electronic and plastic waste, which supports the vision of a sustainable and green world.

## 1. Introduction

HMIs enable the end-user to interact with the electronic devices in a facile way [1,2,3,4,5]. Market-available, plastic, button-based HMIs are neither environmentally friendly nor disposable. These plastic button HMIs are not compact, which makes them difficult to integrate with modern-day thin electronics and are fabricated by polluting processes and materials. In recent years, electronic waste has been generating a major chunk of plastic waste, about 20% [6]. The harmful additives in plastic waste cause environmental and health issues during recycling. Paper-based recyclable green HMIs are the alternative to plastic HMIs and have the potential to contribute to developments in intelligent labels, touch interactive displays, wearable systems, use-and-throw electronics, and smart packaging. To integrate additional functionalities in the HMIs, many efforts have been made previously. For example, foldable sensors have been developed to be utilized as touch sensors for HMIs [7,8,9,10,11,12,13,14]. The development of foldable electronics is primarily done on flexible substrates such as polyethylene terephthalate (PET), polyimide (PI), and polydimethylsiloxane (PDMS) [15,16,17,18,19,20,21]. The degradability of these polymer materials is a major issue that has harmful impacts on the environment. Other than on and off buttons, sensors have been reported for touch and no-touch detection as well [22,23,24,25].

HMIs make the interaction between humans and machines facile and easy to use. Over history, HMIs have evolved from electrical and mechanical switches to batch interfaces to command line interfaces and graphic user interfaces. Modern HMIs consist of an array of touch sensors where each sensor controls different functions of the machine. The electrical properties of the sensor change upon touch, and a controller can observe this change in electrical properties; thus, detecting the touch and triggering an action accordingly. Based on the sensing mechanism, there are different types of touch sensors, such as resistive, capacitive, piezo-capacitive, triboelectric, and piezoelectric [26,27,28,29,30,31,32,33,34].

In the existing literature, there are numerous reports on HMIs fabricated using different materials and methods. For example, laser cutting of metalized paper has been used to fabricate touch keypads for cubes and alarmed cardboard boxes [1]. Graphite on cellulose paper-based touch sensors were fabricated to control the on/off operation of the LEDs [35]; this approach is eco-friendly, although the four sensors were fabricated using unreliable and unstable crocodile pins. Silver ink-based sensors were printed on PDMS to develop a 2 × 2 array of transparent sensors to control the on/off operation of four LEDs through wires [36]. A touchpad consisting of ten touch sensors fabricated on paper through hydrothermal growth of a zinc oxide nanowire was reported to control the on/off operation of the LEDs [37]. In another study, a touchpad was fabricated by direct writing of silver nanowire ink on the paper-based substrate to control the on/off operation of the LEDs [38]. However, most of the existing studies are based on conductive inks and materials such as ZnO nanowires, silver nanoparticle ink, and carbon nanotubes [39,40,41,42]. These inks and materials cannot be labeled as green materials because preparing these materials includes multistep, solvent-based, costly procedures and diffusion of toxic, hazardous chemicals. These issues limit the use of conductive inks to develop touch keypads for environmentally friendly and disposable applications. The fabrication processes involved in developing conventional electronics also involve chemical and gaseous procedures causing greenhouse emissions and are not environmentally friendly [35]. Most of the reported flexible touch sensor-based keypads are fabricated using processes such as etching, lithography, and direct printing, followed by sintering and heating to achieve higher conductivity [1,34,36,37]. Factors such as the use of solvent-based materials in the fabrication processes, cost, and tools required for fabrication result in uneconomical sensors as well as environmental hazards. On the other hand, the graphite-based touch sensors reported in the existing literature only detect touch and no-touch conditions [35]. Therefore, there is a need for green, electronics-based intelligent HMIs that can perform additional functionalities. The most basic HMI form detects touch and no-touch conditions only, whereas intelligent HMIs can provide control, monitoring, and visualization between a human and a machine. The biggest advantage of an intelligent HMI is its user-friendliness. The graphical interface contains color coding that allows for easy identification, as well as pictures and icons allowing for fast recognition, easing the problems of language barriers. This not only reduces the chances of error during application, such as in production plants, but also can reduce production costs by providing efficient control.

Here, we report a pressure-sensitive capacitive, touch sensor-based, green, intelligent HMI. The touch sensors were fabricated from facile and green materials, such as paper and graphite pencils, using the simplest fabrication technique of direct writing of graphite-on-paper. The IDC touch sensor design was optimized by analyzing the physical dimensions of the sensors, such as the width, length, number of electrodes, and spacing between the electrodes. The design was also optimized to best fit the human fingers for pressure-based touch sensing. Six IDC sensors were fabricated next to each other to make a touch keypad that functions as an HMI to control the different actions of a robotic arm. The sensors were tested for touch conditions such as usual-force touch, high-force touch, and no touch. A human touching the sensor changes the capacitance of the specific sensor of the HMI. The controller detects the change in capacitance and turns on the motor associated with the respective touch sensor. We have defined two threshold values of capacitances to differentiate between high-pressure and low-pressure touches. The sensors exhibit a good sensitivity for all three conditions: no touch, low-force touch, and high-force touch. The ability to differentiate between low- and high-pressure touches makes the CPS touch keypad an intelligent HMI. In the current work, we have employed capacitive touch sensors because of their simple design and fabrication, good stability, and low-cost. Furthermore, flexible CPS sensors have many advantages over the market-available, stiff pressure sensors because of their low weight, flexibility, and fast response. Flexible CPS sensors also have shown great potential in wearable devices, e-skins, and soft robotics.

## 2. Materials and Methods

### 2.1. Materials

A standard 80-gram paper with about 100 µm thickness was purchased from a local stationary store. The paper was used as a substrate to fabricate the IDC sensor-based touch keypad. An 8B pencil by MONO with a 90 ± 2% graphite-to-clay content was purchased from a local stationary store to fabricate the graphite film electrodes for the touch sensor-based keypad [34]. An ultrathin sheet, known as Cling, was also purchased locally from a grocery store, which was utilized to protect the graphite sensors from performance degradation because of human finger rub and humidity.

### 2.2. Design and Fabrication

Capacitive sensors can be of two types based on the structure of the electrodes: either in-plane or parallel plates. The lower repeatability and sensitivity of the parallel plate capacitive sensors limit their use for practical application [1]. The in-plane capacitive structure, also called IDC structure, is based on electrode design. Both the capacitor electrodes in the IDC scheme are on the same substrate side. One or more than one pair of capacitor electrodes are available to detect the human finger touch. The IDC sensor exhibits low base values but a higher sensitivity towards human touch, with a 3–4 times change in capacitance under touch and no-touch conditions.

**Figure 1:**
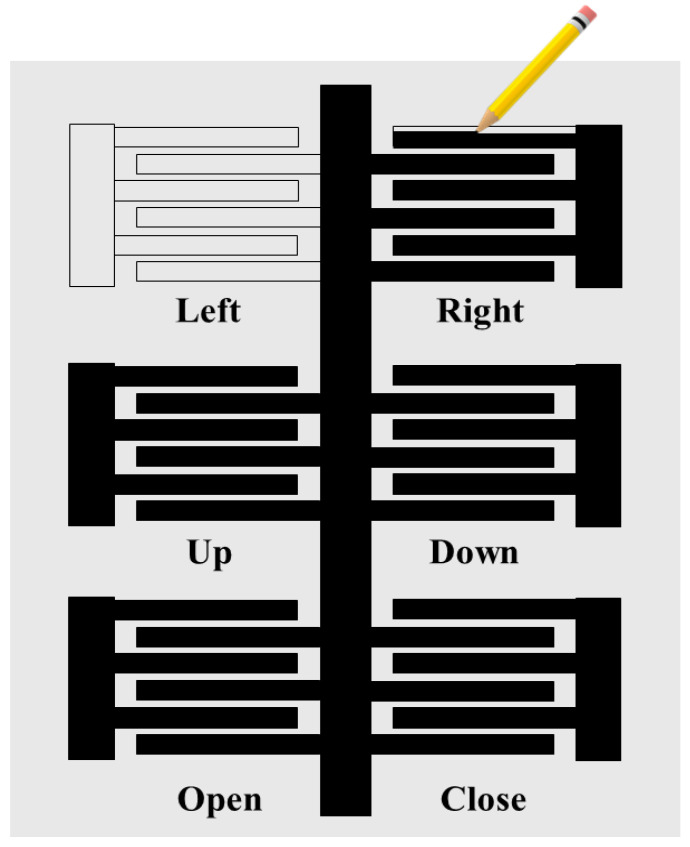
GOP-based fabrication process to fabricate the CPS touch keypad-based HMI.

The IDC sensor was designed in MS Word by optimizing parameters such as the length, width, number of sensor fringes, and gap between the fringes. The optimization parameters involve the number of electrode fingers, finger width, finger overlap length, and spacing between the electrode fingers. The designed single sensors were arranged in an array of six to make an IDC sensor-based touch keypad. The designed keypad was printed on an A4 printer paper using an HP printer. A graphite-on-paper (GOP)-based manual fabrication process was used to fill the hollow IDC touch sensors, as shown in Figure 1. The first layer of the graphite was filled in the printed pattern in one direction and the results were tested. The results obtained from the sensors after the first layer were unreliable and non-repetitive because the graphite layer was inconsistent and had empty spaces on the surface. Consistency in the electric properties of the graphite and thus the sensor’s working was achieved through deposition of the graphite layers in different directions. The next layer of the GOP was deposited perpendicular to the first layer. Six layers of graphite were deposited in the same way in different directions, achieving uniform results from the sensors.

Figure 1 shows the graphite-on-pencil-based fabrication process to fabricate the touch keypad-based HMI. The solvent-free GOP fabrication process utilized in this work does not include any toxic, solvent, or adhesive materials. The performance of the GOP-based fabricated sensors decreased after human finger touch because of rubbing. It was also showing fluctuations due to a change in environmental factors, such as humidity. To eliminate the performance decrement and fluctuation issues, the sensor was passivated using a removable and transparent ultra-thin Cling sheet. The addition of the Cling sheet did not affect the overall performance of the sensors.

## 3. Experimental Section

**Figure 2:**
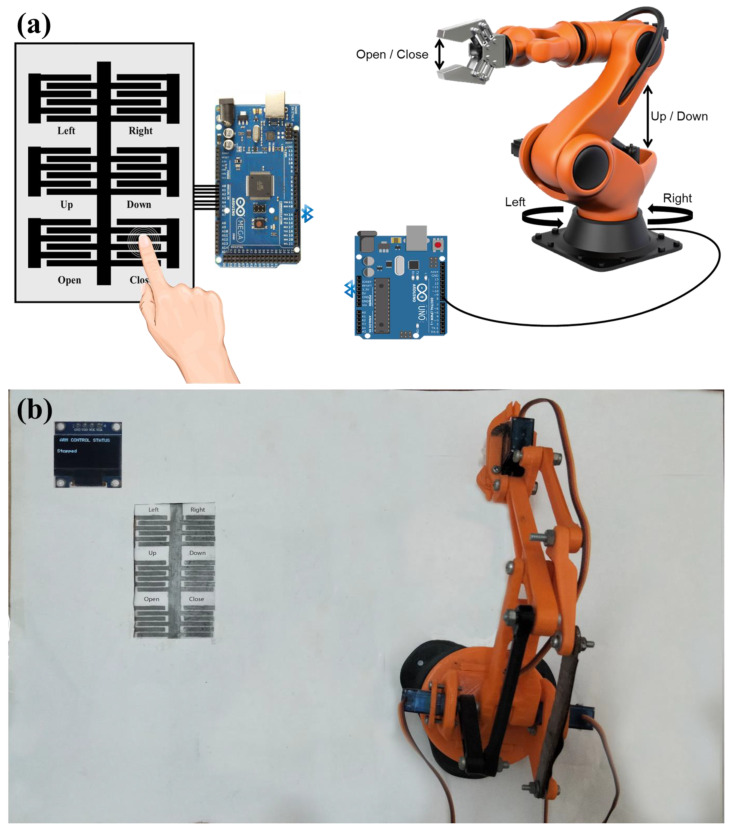
IDC touch keypad for an intelligent HMI. (**a**) Graphical illustration includes the keypad-based HMI integrated with an Arduino communicating through Bluetooth, with the Arduino attached to a robotic arm to control its movements. (**b**) Picture of the setup, including the graphite-on-paper-based IDC touch keypad and a robotic arm to be controlled with the HMI.

The fabricated touch keypad-based HMI was connected with the analog input ports of Arduino Mega to measure the capacitive response of the sensors. Arduino has an internal resistance that combines with the capacitive sensors to make an RC circuit. The Arduino detects the change in capacitance by measuring the voltage across the capacitive sensors in the RC circuit. A 3D-printed robotic arm with three SG90 servo motors was developed to be controlled by the graphite touch keypad. Six touch sensors would control three types of movements of the robotic arm. One of the servo motors was installed at the base of the robotic arm to achieve its left/right 180° rotations and controlled by the top two sensors, as shown in Figure 2a. The second motor is present at the robotic arm’s center joint to provide the chunk’s up/down movements. The third motor allows the opening and closing of the chunk of the arm. The robotic arm is connected to an Arduino and all the motors of the robotic arm are controlled through this Arduino. A picture of the experimental setup includes the robotic arm and HMI, as shown in Figure 2b. The HMI was connected wirelessly with the robotic arm via Bluetooth communication between the HMI Arduino and the robotic arm’s motors controlling the Arduino. The Bluetooth communication between the two Arduino processors was achieved through the HC05 Bluetooth modules. The sensor’s response to the human touch on the keypad was communicated to the respective motor of the robotic arm. The sensor would detect no touch, low-force touch, or high-force touch and the Arduino would communicate it to the robotic arm to control the operation and speed of the different motors in accordance with the touch input. A low-force touch would result in the slow speed operation of the respective motor while a high-force touch would cause the respective motor to operate at a high speed, which makes our HMI an intelligent HMI.

## 4. Results and Discussion

**Figure 3:**
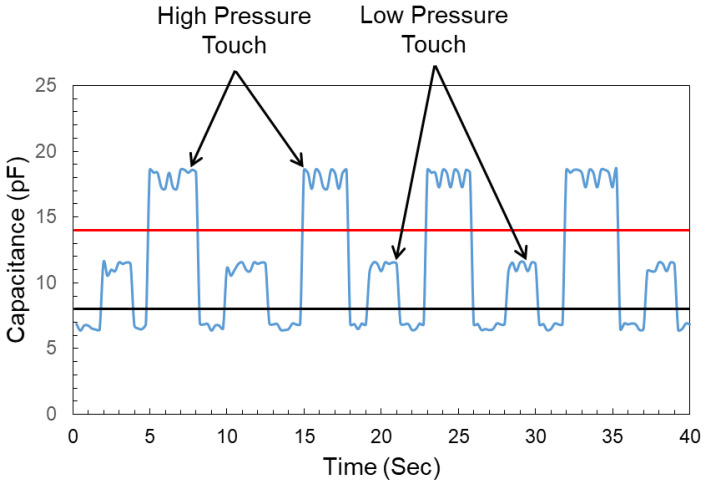
Single sensor response to no touch, low-force touch, and high-force touch conditions.

Capacitive touch sensors were utilized in this work because of their high repeatability, sensitivity, and low cost in developing CPS, keypad-based HMIs. Each keypad sensor consists of six fingers of 20 mm in length and 2 mm in width. The spacing between the fingers is 1 mm. All the keypad sensors were tested rigorously to no touch, low-force touch, and high-force touch to decide the thresholds for all three conditions. The change in the capacitance with low- and high-force touches was differentiable in comparison with the no-touch condition. The lines at 8 pF and 14 pF show the threshold to register the low-force touch and high-force touch consecutively. A single sensor response towards no-touch, low-pressure touch, and high-pressure touch is shown in Figure 3.

**Figure 4:**
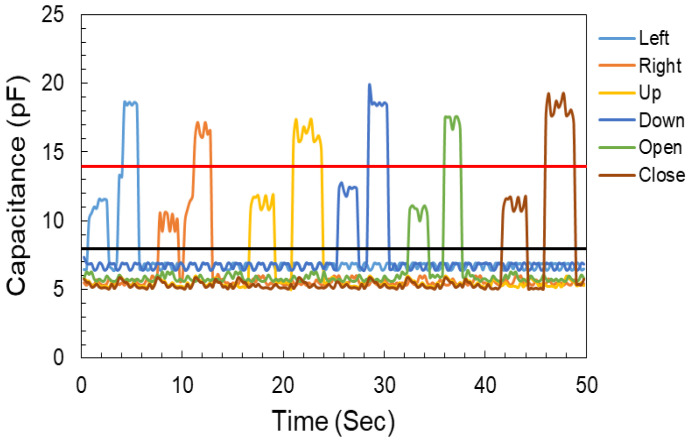
CPS, keypad-based HMI capacitance response to no touch, low-pressure touch, and high-pressure touch conditions.

Six sensors were arrayed in a sequence to design a keypad, as shown in Figure 1. The arrayed sensors were named according to the respective controls of the robotic arm: the Left and Right sensors controlled the left/right movements of the base of the robotic arm; Up and Down sensors controlled the up/down movement of the chunk of the robotic arm; and Open/Close sensors controlled the open/close operation of the chunk. The capacitive response of all the sensors of the keypad-based HMI under no-touch, low-pressure touch, and high-pressure touch is shown in Figure 4. Each sensor was touched with high as well as low pressure, which resulted in a change in capacitance of the sensor. The controller detects this change in capacitance and triggers an action based on the threshold values of the capacitances, as shown in Figure 4. The black line at 8 pF shows the threshold to register the touch condition under low force. The red line at 14 pF was a second threshold to detect the high force touch. The high force touch gets registered when the sensor’s response crosses the second threshold.

**Figure 5:**
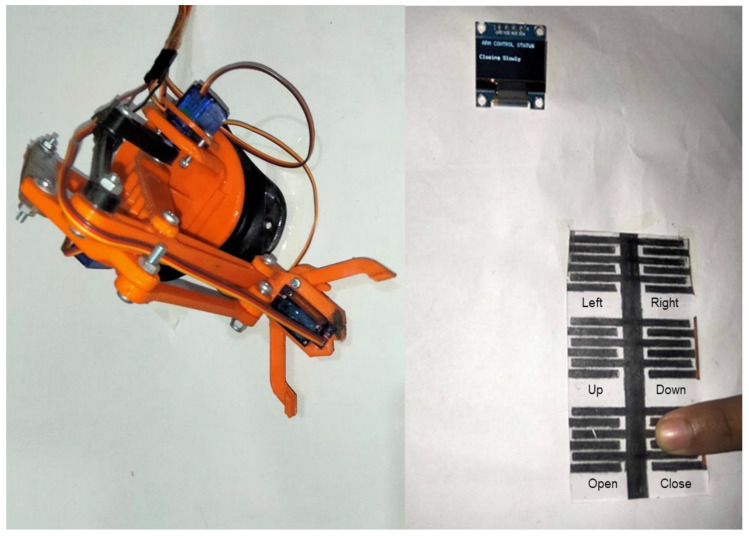
Picture of the CPS, keypad-based HMI integrated with a robotic arm.

The HMI was integrated with a robotic arm to control the 2D movement of its bases, the up/down movement of the chunk, and the open/close movement of its chunk. The speed of the robotic arm’s three motors was linked with the keypad and depended upon the pressure applied to the touch keypad. Six CPS sensors of the touch keypad were linked to the three motors of the robotic arm wirelessly: two sensors (left and right) controlled the 2D base movement in the clock, anti-clockwise; two sensors (up and down) controlled the up and down movement of the chunk; and two sensors (open and close) controlled the opening and closing of the chunk of the robotic arm. No movement was allowed to the robotics arm’s motors under no-touch conditions. Low-pressure touch allowed a 3° movement, while a high-pressure touch allowed a 5° movement to all of the motors of the robotic arm. The high degree of movement allows the fast movement to cover the large movements rapidly, and the low degree of movement allows the low-speed movement to achieve precision. Figure 5 shows the robotic arm, integrated with the CPS, keypad-based HMI wirelessly, and an example of a finger touch on the Close sensor to close the chunk slowly.

**Figure 6:**
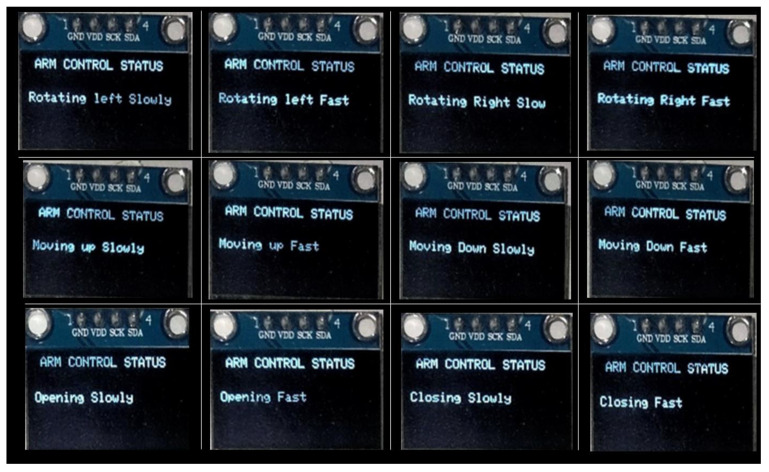
Status of the robotic arm motors with reference to the force applied (low or high) on the respective touch sensor of the CPS, keypad-based HMI.

An OLED was connected to the system to show the status of the sensors under no touch, low-force touch, or high-force touch. In addition, a message regarding low- and high-force touch was displayed using an OLED, to guide the user. The user would be informed about the sensor on which a touch was detected and whether the respective motor is moving fast or slow in response to the touch. Figure 6 displays all possible messages on the OLED. These pictures were taken during the keypad testing to show the status of the motors, either moving the arm slowly or rapidly. The top row of Figure 6 displays the status of the motor controlling the left/right movement of the robotic arm. In response to the input provided by the user, the motor can move the arm left or right at a slow or fast speed. All possible statuses of this motor are shown in the top row. The middle row displays the status of the up/down motor that can move in an upward or downward direction at a slow or fast speed. The bottom row represents the status of the jaw, which can open or close at a slow or fast speed, in accordance with the input provided by the user, where a slow movement is due to a low-force touch and fast movement is due to a high-force touch. The presented results show that the CPS touch keypad fabricated from green and facile materials and fabrication techniques are capable enough to control the machines intelligently based on the touch pressure. The obtained results are sensitive to the paper type and the surface roughness of the paper can affect the results. Further, the reported fabrication process is manual and cannot be used for large areas and speedy manufacturing. Future studies can focus on a speedy fabrication process compatible with graphite-based green electronics to achieve high-throughput, large-area fabrication.

## 5. Conclusions

The development of facile and green graphite-on-paper-based fabricated pressure-sensitive touch keypad was explored. The keypad-based HMI exhibits a measurable change in capacitance upon low- and high-pressure touches. The HMI, wirelessly integrated with the robotic arm, controlled the arm’s movements without delay. The sensor system effectively controlled the three motors of the robotic arm at low and high speeds based on the figure touch pressure. The reported keypad-based HMI is reliable, consistent, disposable, and cost-effective. This demonstration of the CPS keypad shows that it can be used in diverse applications in healthcare, soft robotics, wearable systems, displays, and HMIs.

## Data Availability

Data underlying the results presented in this paper is not publicly available at this time but may be obtained from the authors upon reasonable request.
